# Effect of Mutated *ids* Overexpression on IDS Enzyme Activity and Developmental Phenotypes in Zebrafish Embryos: A Valuable Index for Assessing Critical Point-Mutations Associated with Mucopolysaccharidosis Type II Occurrence in Humans

**DOI:** 10.3390/diagnostics10100854

**Published:** 2020-10-21

**Authors:** Cheng-Yung Lin, Hsiang-Yu Lin, Chih-Kuang Chuang, Po-Hsiang Zhang, Ru-Yi Tu, Shuan-Pei Lin, Huai-Jen Tsai

**Affiliations:** 1 Institute of Biomedical Sciences, MacKay Medical College, New Taipei City 25245, Taiwan; cylin@mmc.edu.tw (C.-Y.L.); lxc46199@ms37.hinet.net (H.-Y.L.); wzmf173814@gmail.com (P.-H.Z.); 2 Department of Medicine, MacKay Medical College, New Taipei City 25245, Taiwan; 3 Department of Pediatrics, MacKay Memorial Hospital, Taipei 10449, Taiwan; 4 Department of Medical Research, MacKay Memorial Hospital, New Taipei City 25160, Taiwan; mmhcck@gmail.com (C.-K.C.); likemaruko@hotmail.com (R.-Y.T.); 5 MacKay Junior College of Medicine, Nursing and Management, Taipei 11260, Taiwan; 6 Department of Medical Research, China Medical University Hospital, China Medical University, Taichung 40402, Taiwan; 7 College of Medicine, Fu-Jen Catholic University, Taipei 24205, Taiwan; 8 Department of Infant and Child Care, National Taipei University of Nursing and Health Sciences, Taipei 11219, Taiwan

**Keywords:** enzyme activity, IDS, mucopolysaccharidosis type II, overexpression, phenotype, zebrafish

## Abstract

Mucopolysaccharidosis type II (MPS II) is an X-linked disorder resulting from a deficiency in iduronate 2-sulfatase (IDS), which is reported to be caused by gene mutations in the iduronate 2-sulfatase (IDS) gene. Many IDS mutation sites have not yet had their causal relationship with MPS II characterized. We employed a gain-of-function strategy whereby we microinjected different mutated zebrafish *ids* (z-*ids*) mRNAs corresponded to human *IDS* gene into zebrafish embryos, and then measured their total IDS enzymatic activity and observed the occurrence of defective phenotypes during embryonic development. We examined three known mutation sites for human IDS genes (h-IDS) associated with MPS II symptoms, including h-IDS-P86L, -S333L and -R468W, which corresponded to z-*ids*-P80L, -S327L and -R454W. When these three mutated z-*ids* mRNAs were overexpressed in zebrafish embryos, the IDS enzymatic activity of the total proteins extracted from the injected embryos was not increased compared with the endogenous IDS of the untreated embryos, which suggests that the IDS enzymatic activity of these three mutated z-*ids* was totally lost, as expected. Additionally, we observed defective phenotypes in these injected embryos, resulting from the failed IDS enzyme breakdown, which, in turn, has a dominant negative effect on the endogenous wild-type IDS function. These phenotypes were similar to the clinical symptoms observed in MPS II pathogenesis. We further studied six uncharacterized IDS mutation sites as identified by the Taiwanese MPS newborn screening programs. We propose a novel IDS enzyme activity assay combined with phenotypic observation in zebrafish embryos, as an alternative platform for quickly providing a valuable index for preliminarily assessment of any identified IDS point mutation gene that has not yet been characterized, in the context of its role in MPS II development.

## 1. Introduction

Mucopolysaccharidosis type II (MPS II), or Hunter syndrome, is a lysosomal storage disorder, characterized by a lack of the specific enzymes that break down fats or sugars. More specifically, it is an X-linked disorder resulting from a deficiency in iduronate 2-sulfatase (IDS), which catalyzes the hydrolysis of the 2-sulphate group of dermatan sulfate and heparan sulfate. An IDS gene mutation typically underlies the abnormal accumulation of these two glycosaminoglycans (GAGs), resulting in dysfunction of most organ-systems, including the brain, heart, liver, central nervous system, and skeletal system. In the majority of patients [1], as seen in Asia and Taiwan [2,3,4,5], the clinical forms of MPS II are either mild or severe, depending on the age at onset and the clinical manifestations [6]. Patients with the mild form of MPS II usually have attenuated somatic complications without mental disability. Patients with the severe form of MPS II show early somatic abnormalities combined with skeletal deformities, hepatosplenomegaly, and progressive cardiopulmonary deterioration. Consequently, neurological damage presents progressively and prominently as developmental delay and intellectual disability, often concomitant with neurodegeneration.

Although the manifestations of MPS II are progressive and serious, infants are typically born without any easily identifiable clinical symptoms. Depending on the specific type and severity of the disease, symptoms typically present within the first 2–4 years of life and progress rapidly thereafter [6]. Thus, in this 2–4 year window, signs and symptoms of MPS II can be missed, leading to a delay in diagnosis and treatment. As a result of new molecular biology techniques in combination with newborn screening [7,8,9], at least 658 mutations in the IDS gene have so far been identified [Human Gene Mutation Database (HGMD) Professional 2019.1], including missense/nonsense mutations, deletions, insertions, and rearrangements [1]. Although some mutation sites have been identified from clinical symptoms [9], most IDS mutations have not been clearly characterized to determine whether they are necessary and sufficient to cause MPS II in humans. Therefore, it would be beneficial to develop a fast but effective platform that can reliably assess which mutated nucleotides of IDS are strongly associated with MPS II occurrence.

The current study reports a quick method of screening for deficiency or malfunctioning of the lysosomal IDS enzyme encoded by a specific mutated gene, which leads to abnormal accumulation of GAGs. To accomplish this, we used zebrafish (*Dario rerio*) as our model animal. Two strategies are most commonly used to determine the biological function of test genes, including loss- and gain-of-function. To carry out the loss-of-function approach, gene knockdown by antisense oligonucleotide morpholino (MO) is typically performed. For example, Moro et al. knocked down zebrafish *ids* (z-*ids*) by injection of an antisense MO oligonucleotide specific for *ids* (z-*ids*-MO) into zebrafish embryos [10]. This resulted in severe developmental defects, including the disruption of body axis organization and the malformation of craniofacial cartilage. Furthermore, Costa et al. reported that a defective atrioventricular valve, similar to MPS II valve disease, was observed in z-*ids*-MO-injected zebrafish embryos [11]. They demonstrated that loss of z-*ids* function caused defective signaling of early Sonic Hedgehog and Wnt/b-catenin, leading to aberrant heart development and atrioventricular valve malformation in zebrafish embryos. Sometimes however, off-target effects, side effects, redundancy, and toxicity derived from MO-injected embryos are reported [12]. We have noticed that MO tends to block the translation of mRNA, reducing the total amount of target protein in zebrafish embryos and that this in turn, results in complete loss of enzymatic activity in the target protein. It should be noted, however, that the amount of IDS in MPS II patients is not necessarily close to zero. For example, a patient with 15% IDS enzyme activity can still suffer from MPS II [9]. Many mutation points on human IDS have been identified [9], indicating that some IDSs encoded by mutated IDS are composed of a full-length IDS polypeptide, but with mutated amino acid residue(s) that result in no, partial, or even intact function of the resultant IDS.

Recently, Bellesso et al. applied CRISPR/Cas9 technology to generate a transgenic zebrafish line possessing a five-base-pair deletion from nucleotide 241 to 245 of z-*ids,* resulting in a premature stop codon at the 118th amino acid and producing a truncated form of z-IDS in cells. They found that loss of z-IDS function in the early developmental stage decreased Fgf signaling, which negatively affected bone development at the later stages [13]. Although CRISPR/Cas9 technology can generate a transgenic line with a single point mutation in the *ids* gene, the procedures are complicated and it takes a relatively long time to obtain lines to study.

Based on the studies described above, it can be concluded that zebrafish are a good animal model with which to study MPS. However, in the current study, we switched the evaluation strategy to the gain-of-function approach in transgenic zebrafish embryos because mRNA is much easier, cheaper, and more convenient to prepare in a lab compared with antisense oligonucleotide MO synthesized by a specific company. In brief, the current study used the gain-of-function approach to build an assay platform to determine the effect of different mutated *ids* nucleotides on both IDS enzymatic activity and the normal development of zebrafish embryos. With this two-pronged strategy, which examined transgenic zebrafish embryos overexpressing mutated IDS, we aimed to quickly evaluate which point mutations were critical to the occurrence of MPS II.

## 2. Materials and Methods

### 2.1. Ethics Statement

The MacKay Memorial Hospital Institutional Animal Care and Use Committee (IACUC) reviewed and approved the protocol described below (MMH-A-S-107-51), approved on 1 August 2019.

### 2.2. Fish Embryos

The wild-type zebrafish AB strain (University of Oregon, Eugene, OR, USA) was used. The culture conditions, embryo stage, egg production, and egg collection were as described previously [14]. The morphological phenotypes of the embryos were observed under a fluorescent stereomicroscope (MZ FLIII, Leica, Wetzlar, Germany).

### 2.3. Knockdown Microinjection of Zebrafish Embryos

The antisense MO (Gene-Tools) used to specifically knock down the translation of zebrafish *ids* mRNA (z-*ids*-MO) was prepared at a stock concentration of 1 mM. Each injection into one- to two-cell-stage embryos was 2.3 nL include 12 ng MO.

### 2.4. Overexpression of ids mRNA

Capped mRNAs of human h-*IDS* and zebrafish z-*ids* and their different mutated forms of *ids* were synthesized according to the manufacturer’s protocol (Epicentre). The synthesized *ids* mRNAs were diluted to 44-, 88-, 132-, and 176-ng/µL with double-distilled water. Each injection into one- to two-cell-stage embryos was 2.3 nL.

### 2.5. Cartilage Staining

Alcian blue staining was performed as described by Lin et al. with some modifications [15]. Embryos at five days-postfertilization (dpf) were anesthetized using 0.02% buffered tricaine (Sigma-Aldrich, St. Louis, MO, USA) and fixed overnight in 4% PFA at 4 °C. After washing with PBS, embryos were stained overnight in 0.1% Alcian blue, which was dissolved in acidic ethanol (70% ethanol, 5% concentrated hydrochloric acid), then washed extensively in acidic ethanol, dehydrated, and stored in 80% glycerol. For better exposure of the cartilage elements, embryos were digested with 0.02% trypsin.

### 2.6. Plasmid Construction

**Figure A1:**
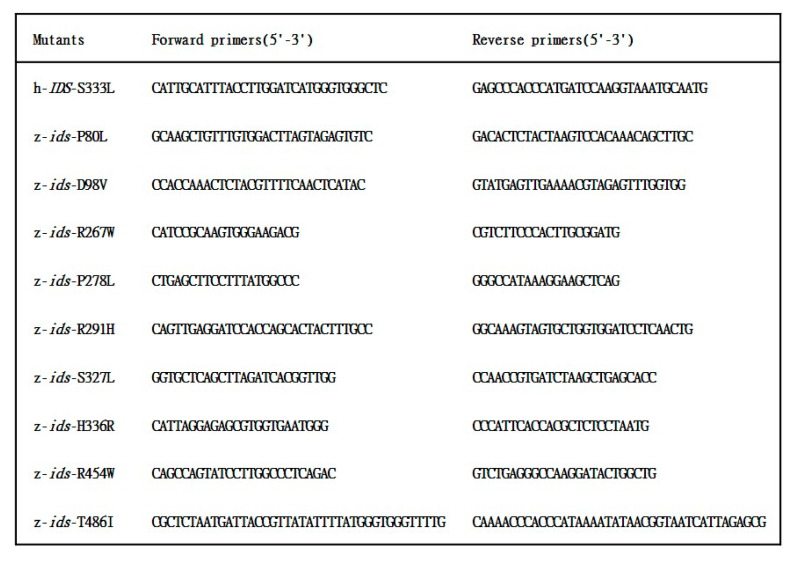
Primers used for site-directed mutagenesis.

The coding region of human IDS cDNA (L40586.1) and zebrafish *ids* cDNA (NM_001080068.1) tagged with the reporter FLAG peptide were engineered into plasmid pCS2^+^ vectors to generate pCS2-h-IDS-flag and pCS2-z-IDS-flag plasmids, respectively. Various point-mutated forms of h-IDS and z-*ids* were obtained by PCR mutagenesis using primers as listed in Appendix A Figure A1.

### 2.7. Western Blot Analysis

Total protein was extracted from embryos and analyzed on 10% SDS-PAGE followed by western blot analysis according to the procedures described by Lin et al. [16], except that the yolk was removed by deyolking buffer (55 mM NaCl, 1.8 mM KCl and 1.25 mM NaHCO_3_) and antibodies against Flag (Abcam, Cambridge, UK; 1:5000 dilution), α-tubulin (Sigma-Aldrich, St. Louis, MO, USA; 1:5000 dilution), mouse-HRP (Santa Cruz, Santa Cruz, CA, USA; 1:5000 dilution), and rabbit-HRP (Santa Cruz; 1:5000 dilution) were used.

### 2.8. IDS Enzyme Activity

A modified version of the protocol described by Moro et al. and Lin et al. was used to measure the enzymatic activity of IDS from proteins extracted from zebrafish embryos [9,10]. Briefly, 15 μg of extracted proteins were dissolved in 10 µl whole-cell extraction buffer (20 mM HEPES, 0.2 M NaCl, 0.5% TritonX100, 20% glycerol and 1 mM EDTA), added to 20 μL MPS II substrate (0.1 M sodium acetic acid buffer, pH 5.0, containing 10 mM lead acetate), and incubated at 37 °C for 4 h. Then, 20 μL Pi/Ci buffer (0.2 M NA_2_HPO_4_/0.1 M citric-acid buffer, pH 4.5, containing 0.02% Na-azide) and 10 μL α-L-Iduronidase human (1 µg/mL) were added to each sample and incubated at 37 °C for a further 24 h. The reaction was stopped by adding 200 μL stop buffer (0.5 M NaHCO_3_/0.5 M Na_2_CO_3_, pH 10.7, containing 0.025% Triton X-100). The ratio of 365/450 nm excitation/emission on a 96-well plate was counted using a Victor Nivo TM Multilabel Counter Fluorometer (PerkinElmer, Inc., Waltham, MA, USA). IDS enzyme activity was defined as the nanomoles of substrate hydrolyzed in 4 h/mg of protein (nmol/mg protein/4 h). Compared to the IDS enzyme activity of the untreated control group, which was set as 1, the relative enzymatic activity of total IDS obtained from the zebrafish embryos injected with various mutated IDS mRNAs were determined.

## 3. Results

### 3.1. Overexpression of Zebrafish ids mRNA in Fertilized Eggs Increases the Enzymatic Activity of IDS in Zebrafish Embryos

**Figure 1:**
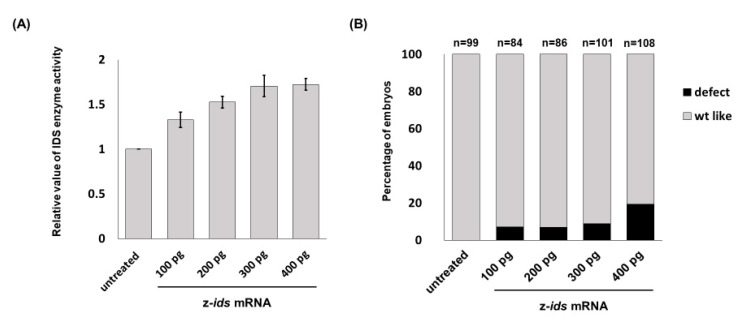
Enzymatic activity of iduronate 2-sulfatase (IDS) and the occurrence rate of developmental defects in zebrafish embryos injected with different concentrations of z-*ids* mRNAs. (**A**) Zebrafish embryos at one-cell stage were injected with 100-, 200-, 300-, or 400-pg of z-*ids* mRNAs, followed by detection of IDS enzymatic activity at 48 hpf. The IDS enzymatic activity of the untreated control group was normalized as 1 for comparison with the embryos injected with different dosages of z-*ids* mRNAs. (**B**) The occurrence rates (in percentage) of defective phenotypes among zebrafish embryos injected with 100-, 200-, 300-, and 400-pg, z-*ids* mRNAs were calculated when embryos were examined at 5 dpf.

Zebrafish embryos served as an in vivo platform to evaluate whether mutated IDS contributes to MPS II and if so, to what degree. To make this determination, we microinjected *ids* mRNA into one-cell fertilized zebrafish eggs, followed by quantification of IDS enzymatic activity at 48 h post-fertilization (hpf), and observed the phenotypes during embryonic development. The endogenous enzymatic activity of IDS (z-IDS) in the untreated control zebrafish embryos was set as 1. Against this, we compared the z-IDS enzymatic activity of the zebrafish embryos injected with z-*ids* mRNA (300 pg/embryo), and we found it to be significantly increased at 1.71 ± 0.12 (Figure 1). These findings suggest that injection of z-*ids* mRNA increased the total amount of z-IDS in the zebrafish embryos, leading to increased z-IDS enzymatic activity.

### 3.2. Zebrafish Embryos Injected with h-IDS mRNA Display a Change in IDS Enzymatic Activity

**Figure 2:**
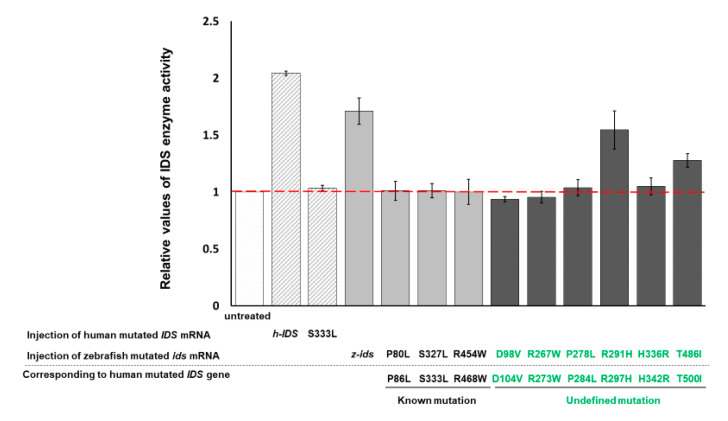
Effect of mutated *ids* mRNA overexpression on the IDS enzymatic activity of zebrafish embryos. Serving as a control group, the IDS enzymatic activity of the untreated zebrafish embryos at 48 hpf was quantified and normalized as 1 for comparison with the relative IDS activity obtained from the other experimental groups. Zebrafish embryos at one-cell stage were injected with 300-pg human IDS mRNA (h-IDS), zebrafish *ids* mRNA (z-*ids)*, and various point-mutated z-*ids* mRNAs, as indicated. Each point-mutated z-*ids* corresponding to that of the h-IDS was listed below the black dotted line.

In a parallel experiment, the enzymatic activity of endogenous z-IDS in untreated control zebrafish embryos at 48 hpf was again set as 1. This time, total IDS enzymatic activity of the zebrafish embryos injected with h-IDS mRNA (300 pg/embryo) significantly increased to 2.04 ± 0.01 (Figure 2). Next, we examined the IDS enzymatic activity of zebrafish embryos injected with human mutated IDS mRNA at 333 nt (h-IDS-S333L) (300 pg/embryo) and found that total IDS enzymatic activity remained unchanged at 1.03 ± 0.02 (Figure 2), suggesting that the enzymatic activity of mutant h-IDS-S333L did not contribute to the total IDS enzymatic activity extracted from zebrafish embryos. These results suggest that h-IDS-S333L lost its enzyme activity, and failed to exert any additional effect on z-IDS or enhance z-IDS endogenous enzyme activity.

From these findings we draw three conclusions. First, the h-IDS protein encoded by injected *h*-IDS mRNA is functional and presents enzymatic activity. In contrast, the h-IDS-S333L mutated protein encoded by h-IDS-S333L mRNA malfunctions and loses its enzyme activity. Second, the enzymatic activity of IDS extracted from h-IDS-injected zebrafish embryos exhibited an additive enzymatic effect from both z-IDS and h-IDS, whereas the enzymatic activity of IDS extracted from h-IDS-S333L-injected embryos did not. Third, the enzyme activity of h-IDS and its mutated form can be monitored by measuring the enzyme activity of total IDS in zebrafish embryos after injection.

In summary, these results suggest that mutation of IDS at 333 amino acid completely abolishes the enzymatic activity of h-IDS-S333L. Therefore, we concluded that overexpression of h-IDS and its mutant mRNAs in zebrafish embryos using the gain-of-function approach is a feasible strategy for quickly determining whether mutated IDS can or cannot affect the enzymatic activity of total IDS in the injected embryos. If IDS enzyme activity was not added, then the pathogenicity of MPS II could be reasonably deduced.

### 3.3. IDS Enzyme Activity Remains Unchanged in Zebrafish Embryos Injected with Three Zebrafish Mutated ids mRNAs Corresponding to Three Known Human Mutated ids

We have confirmed that human h-IDS can be replaced by zebrafish z-IDS to study the enzymatic behavior of mutated IDS in zebrafish embryos. Therefore, we further selected three mutation sites of h-IDS: h-IDS-P86L, -S333L and -R468W, all of which are reported in human MPS II pathogenesis [17,18]. These h-IDS mutation sites correspond to the mutation sites of z-IDS, namely z-IDS-P80L, -S327L and -R454W, respectively. When these three mutant z-*ids* mRNAs were individually injected into the zebrafish embryos, the relative enzymatic activity of total IDS in the embryos was 1.01 ± 0.08, 1.01 ± 0.06 and 1.00 ± 0.11, respectively, compared to the enzymatic activity of IDS in the untreated control group, which was set as 1 (Figure 2). Therefore, exogenous mutant z-*ids* mRNAs, corresponding to known mutated human IDS, as reported in MPS II patients, did not exhibit z-IDS enzyme activity significantly higher than that of the untreated control embryos. This means that the enzyme activity of total IDS was not increased by the overexpression of all three mutant z-*ids* mRNAs, as examined in zebrafish embryos, indicating that the h-IDS-P86L, -S333L and -R468W h-IDS mutants completely lost their IDS activity, resulting in malfunctional h-IDS. Taken together, we propose that IDS between zebrafish and human is functionally conservative, leading to speculation that zebrafish and humans might have similar functional IDS domains, owing to the loss of enzymatic activity arising from corresponding point mutations between them.

### 3.4. IDS Enzyme Activity of Embryos Injected with Six Zebrafish Mutated ids mRNAs Corresponding to Six Uncharacterized Human Mutated ids

Next, we investigated the enzyme activity of IDS in six uncharacterized mutated h-IDS: h-IDS-D104V, -R273W, -P284L, -R297H, -H342R and -T500I, which were obtained from the Taiwanese MPS Newborn Screening Project. These h-IDS mutation sites correspond to the z-IDS at D98V, -R267W, -P278L, -R291H, -H336R and -T486I, respectively. In this experiment, six mutant z-*ids* mRNAs were injected into zebrafish embryos individually. Compared to the enzymatic activity of IDS in the untreated control group, which was set as 1, the relative enzymatic activity of total IDS in the embryos injected with the six mutated z-*ids* mRNAs were as follows: z-IDS-D98V: 0.94 ± 0.02, -R267W: 0.95 ± 0.05, -P278L: 1.04 ± 0.07, -R291H: 1.55 ± 0.17, -H336R: 1.05 ± 0.07, and -T486I: 1.28 ± 0.06 (Figure 2). In this experiment, overexpression of z-IDS-D98V, -P260R, -R267W, -P278L, and -H336R in the embryos did not increase the enzyme activity of the total IDS examined from the embryos, suggesting that the IDS enzyme activity of these six mutants was completely diminished. This conclusion was based on the absence of an additive effect on the total IDS activity in these embryos following the injection of these mutant z-*ids* mRNAs. In contrast, zebrafish embryos overexpressing z-IDS-R291H and -T486I did show an increase in total IDS enzymatic activity due to the additive effect of the exogenous z-IDS-R291H and -T486I proteins, suggesting that the IDS enzyme activity of z-IDS-R291H and -T486I proteins was not completely diminished in these two mutants. Nevertheless, we noticed that the enzyme activity of total IDS from zebrafish embryos overexpressing either z-IDS-R291H or z-IDS-T486I protein was still not as high as that of embryos overexpressing wild-type z-IDS, suggesting that the two mutant z-IDS-R291H and z-IDS-T486I proteins only possess partial enzyme activity compared to that of embryos injected with wild-type z-*ids* mRNA.

### 3.5. Overexpression of Three Zebrafish Mutated ids mRNAs Corresponding to Three Known Human Mutated IDS Affects the Embryonic Development of Zebrafish

**Figure 3:**
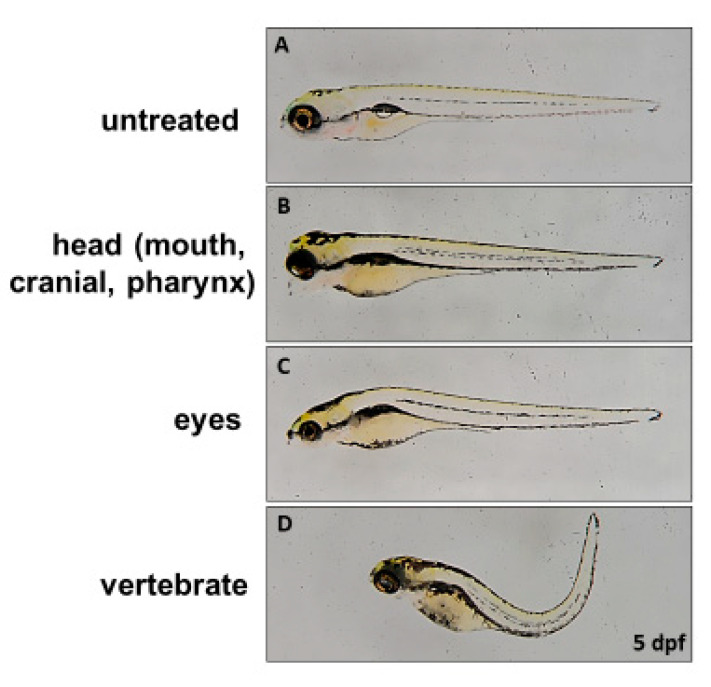
Overexpression of a homologous mutated *ids* found in MPS II interferes with the embryonic development of zebrafish. Embryonic phenotypes at one-cell stage were examined at 5 dpf in zebrafish embryos injected with 300-pg z-*ids*-S327L mRNA, which corresponds to h-IDS-S333L. (**A**) Untreated embryos; (**B**–**D**); embryos injected with mutant z-*ids*-S327L: (**B**) head defect, including smaller size and malformation of the craniofacial and pharyngeal arch; (**C**) eye defect, including smaller eyes; and (**D**) body axis defect, including shortened somites and curved body axis.

**Figure 4:**
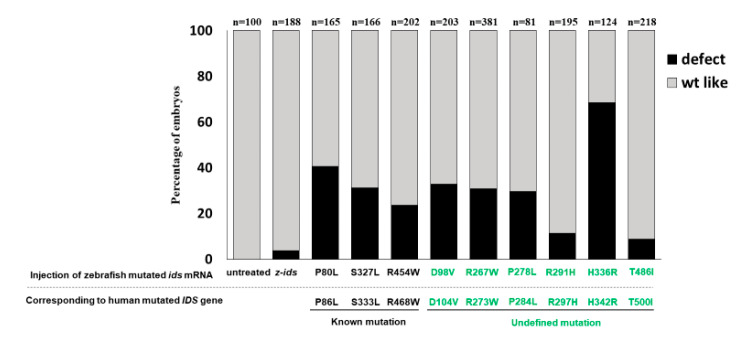
The percentage of defective phenotypes occurring in all examined embryos injected with different mutant forms of z-*ids* mRNAs. Embryonic phenotypes were examined at 5 dpf in the zebrafish embryos injected at one-cell stage with 300-pg zebrafish *ids* mRNAs (z-*ids*), including mutated variants, as indicated. Each mutated *ids* gene of the zebrafish corresponds to a human mutated IDS gene, as listed below. The occurrence rates (in percentages) of the defective phenotypes (marked in dark boxes) and the wild-type-like phenotype (marked in grey) among the total examined embryos injected with different mRNAs were calculated. The total number (n) of embryos studied in each injection group was indicated on the top of each column.

We next turned our attention to the effect of mutated *ids* mRNA overexpression on embryonic development. First, we injected three mutated z-*ids* mRNAs, including z-IDS-P80L, -S327L and -R454W, corresponding to h-IDS-P86L, -S333L and -R468W, respectively, which have been previously reported as MPS II homologous pathogenic sites [17,18]. Compared to the non-injected wild-type at 5 dpf (Figure 3A), three major defective phenotypes were observed in the 5 dpf embryos injected with these three mutated z-*ids* mRNAs: (1) head defect, including smaller size and malformation of the craniofacial and pharyngeal arch (Figure 3B); (2) eye defect, including smaller eyes and brain (Figure 3C); and (3) body axis defect, including shortened somites and curved body (Figure 3D). The ratio of defective development observed in all embryos injected with z-*ids*-WT mRNA, which served as the control group, was ~10% (n = 188), and resulted from physical damage caused by the microinjection (Figure 4). However, the ratio of defective development observed in all embryos injected with the three mutated z-*ids* mRNAs was: 40.6% (n = 165) for z-IDS-P80L; 31.2% (n = 202) for -S327L, and 23.6% (n = 203) for -R454W (Figure 4), suggesting that overexpression of the z-IDS-P80L, -S327L, and -R454W proteins notably increased the rate of embryo abnormality during development.

**Figure 5:**
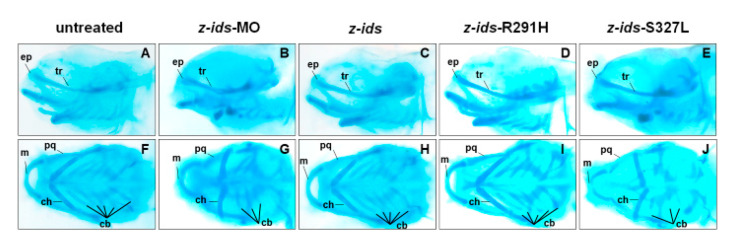
Defective phenotypes of head cartilage during the development of zebrafish embryos injected with different mutant forms of z-*ids* mRNA and z-*ids*-MO. Using Alcian blue staining to detect head cartilage development in zebrafish embryos at 5 dpf. Embryos were injected with the antisense morpholino oligonucleotides (z-*ids*-MO) and different mutant forms of z-*ids* mRNAs, as indicated. (**A**–**E**) Lateral view; (**F**–**J**) Ventral view. (**A**,**F**) Untreated control embryos; (**B**,**G**) z-*ids*-MO-, (**C**,**H**) z-*ids*-, (**D**,**I**) z-*ids*-R291H-, and (**E**,**J**) z-*ids*-S327L-mRNA-injected embryos. cb, ceratobranchial; ch, ceratohyal; ep, ethmoid plate; m, Meckel’s cartilage; pq, palatoquadrate; tr, trabecular plate.

To further confirm the morphology of the craniofacial and pharyngeal arch in detail, we chose z-*ids*-S327L-injected embryos as an example and examined the head cartilage development using Alcian blue staining at 5 dpf. We first used an antisense oligonucleotide MO specific for the knockdown of zebrafish endogenous IDS (z-*ids*-MO), which served as the positive control. Compared to the non-injected control group (Figure 5A,F), the ethmoid plate (ep), and trabecular plate (tr), which support the eye socket of the forehead, were shortened in the z-*ids*-MO-injected group (Figure 5B,G). Not only was the size of the pharyngeal arch cartilage, such as Meckel’s (M), palatoquadrate (pq), ceratohyal (ch), and ceratobranchial (cb) reduced, but also the number of chondrocytes was diminished (Figure 5B,G). Similar to z-*ids*-MO-injected embryos, cartilage development defects were also observed in z-*ids*-S327L-injected embryos (Figure 5E,J).

Conversely, although the ratio of defective development was increased in embryos injected with the three mutated *ids* mRNAs mentioned above, we noticed that their total IDS enzymatic activity did not significantly change, as shown in Figure 3, suggesting that overexpression of these mutated IDS might interfere with normal embryonic development. To explain the increased abnormality rate, we reasoned that these three malfunctioning mutant IDSs likely have a dominant negative effect on normal IDS activity, thus interfering with endogenous z-IDS function in the injected embryos.

These findings strongly support our hypothesis that zebrafish can serve as a model for efficient and rapid in vivo assaying to isolate the critical IDS mutation sites affecting IDS enzyme activity and the development of zebrafish embryos in the context of MPS II.

### 3.6. Overexpression of Six Zebrafish Mutated ids mRNAs Corresponding to Six Uncharacterized Human Mutated IDS Affects the Embryonic Phenotype and IDS Enzymatic Activity of Zebrafish

We have learned that the mutated *ids* genes known to cause MPS II mRNAs could be confirmed through examination of embryonic phenotypes and changes in IDS enzyme activity. Therefore, we next investigated if the overexpression of six point-mutated *ids* mRNAs newly found in Taiwan could affect embryonic phenotype development and the IDS enzymatic activity of injected zebrafish embryos. Our results demonstrated that the ratios of defective embryonic phenotypes observed in all injected embryos were 32.7% (n = 107), 30.9% (n = 81), 29.5% (n = 166), 11.3% (n = 195), 68.5% (n = 124), and 8.7% (n = 218) for z-IDS-D98V, -R267W, -P278L, -R291H, -H336R and -T486I, respectively (Figure 4). Since the occurrence of defective phenotypes was relatively low in z-*ids*-R291H-injected embryos, we selected the z-*ids*-R291H-injected embryos to examine head cartilage development in detail. Unlike the defective phenotypes observed in z-*ids*-MO-injected embryos, no head dyschondroplasia as described in the above section was observed, in either the zebrafish injected with z-*ids*-WT or the zebrafish injected with z-*ids*-R291H at 5-dpf (Figure 5C,D,H,I).

We also examined the IDS enzyme activity of embryos injected with these six point-mutated z-*ids* mRNAs. We found that the embryos injected with z-IDS-R291H and -T486I displayed additive enzymatic activity for z-IDS, while embryos injected with z-IDS-D98V, -R267W, -P278L and -H336R mRNAs did not (Figure 3).

In summary, based on the results of the different overexpressed mutated *ids* mRNAs, we can classify injected embryos into two groups. (a) The z-IDS-D98V-, -R267W, -P278L and -H336R-injected embryos did not display an additive increase in IDS activity, but did present with a higher ratio of developmental defects, suggesting that mutation sites at D98V, R267W, P278L and H336R of IDS may negatively affect normal endogenous IDS enzyme activity, in turn resulting in z-IDS-D98V, -R267W, -P278L and -H336R serving as a dominant negative that interferes with developmental processes. (b) The z-IDS-R291H- and z-IDS-T486I-injected embryos did show an additive increase in IDS enzyme activity, but did not present with a higher ratio of defective embryos, suggesting that IDS with mutations at these sites may slightly impact the normal function of IDS in the injected embryos, possibly because z-IDS-R291H and z-IDS-T486I may contain only partial enzyme activity.

## 4. Discussion

To the best of our knowledge, this is the first report to propose a novel IDS enzyme activity assay that combines phenotypes observed in zebrafish embryos as an alternative platform for quickly providing a valuable index to preliminarily assess any IDS point mutation identified, but not yet characterized, in the context of its role in MPS II occurrence.

### 4.1. The Additive Effect of z-IDS Enzyme Activity in Zebrafish Embryos Can Reflect the Effects of Human h-IDS Mutation Sites on Enzyme Activity

**Figure A2:**
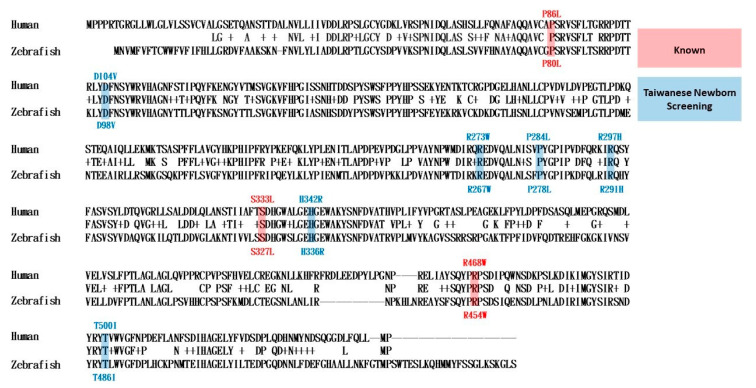
Sequence alignment and conservation between human and zebrafish IDS proteins.

Zebrafish z-IDS and human h-IDS have very similar amino acid sequences, and the original amino acid sequence corresponding to the mutation point is the same (Appendix A Figure A2), therefore, the current study used a zebrafish animal model to provide simple and rapid evaluation and judgment criteria for nine human IDS mutation sites. Firstly, we confirmed that zebrafish z-IDS enzymes had similar functions to human h-IDS enzymes. The same method can be used for enzyme activity detection (Figure 1). H-IDS mRNA was injected into zebrafish embryos to increase h-IDS, which can be added to the endogenous z-IDS enzyme to increase enzyme activity through an additive effect (Figure 3). Secondly, we selected nine human h-IDS mutation sites and mutated the z-IDS at the same homologous site; we then overexpressed the mutant z-IDS in the zebrafish embryos. We found that after the nine mutant z-IDS were overexpressed in vivo, their additive effects on the endogenous z-IDS enzyme activity were the same as that of the nine human mutant h-IDS in vitro enzyme activities [9,17,18]. For example, the blood leukocyte IDS enzyme activity of h-IDS-R297H and h-IDS-T500I in MPS newborn screening were 9.2 and 17.15 µmol/g protein/4 h, respectively [9]. Compared with the reference IDS enzymatic activity reference value of 12.89–131.83 µmol/g protein/4 h [9], the two mutant h-IDS had relatively low IDS enzyme activity. Meanwhile, in zebrafish embryos, the enzyme activity of the overexpressed z-IDS was 1.71 ± 0.12 times higher compared with the untreated group. However, following injection of the same dose of mRNA, the enzyme activity produced by z-IDS-R291H (h-IDS-R297H) and z-IDS-T486I (h-IDS-T500I) was 1.55 ± 0.17 and 1.28 ± 0.06 times higher than the untreated group, respectively. It showed that although the enzyme activity had an additive effect, the increase was not as high as normal z-IDS. It indicated that z-IDS-R291H and -T486I only have partial z-IDS enzyme activity. This phenomenon corresponds well to the results in our human subjects [9].

### 4.2. Overexpression of the Human MPS II Mutant Form IDS Can Interfere with Zebrafish Embryonic Development

Using zebrafish embryos as a functional screening tool for diverse IDS mutation sites has another advantage other than enabling you to detect enzyme activity just using cell lines. The zebrafish model can observe whether the morphological development of the embryo is affected by the overexpression of different mutant forms of z-IDS in a short period of five days. In this study, the injection of z-*ids* mRNA had no effect on the development of zebrafish embryos, which showed that the overexpression of the normal z-IDS protein in the embryo did not interfere with embryonic development. However, after the overexpression of three known human MPS II disease homologous sites (h-IDS-P86L, -S333L and -R468W), corresponding mutant forms of z-IDS (-P80L, -S327L and -R454W), and the injection of the same dose of mRNA to the zebrafish embryos, it was found that a higher proportion of embryos were defective (Figure 5). It was postulated that when there is a high number of mutant forms of z-IDS in the embryo, it will compete with the endogenous normal z-IDS, leading to failure of hydrolyzing dermatan sulfate and heparan sulfate or blocking of normal z-IDS involved in the molecular developmental mechanisms. This negative effect results in defective embryonic development.

### 4.3. Postulating the Relationship between the Mutant Form of IDS and the Development of MPS II Based On the Interference of Zebrafish Embryonic Development

Based on the association between the mutation sites of six new human h-IDS (-D104V, -R273W, -P284L, -R297H, -H342R and -T500I) obtained from the MPS newborn screening programs in Taiwan and the corresponding z-IDS (-D98V, -R267W, -P278L, -R291H, -H336R and -T486I), and the subsequent development of either developmental defects or MPS II, two preliminarily conditions were postulated: (1) Overexpression of z-IDS-D98V, -R267W, -P278L and -H336R, which had no enzyme activity, will affect zebrafish embryo development. This result suggests that medical professionals should closely follow-up these cases, which may be highly correlated with the occurrence of MPS II. In a current case in our hospital, it is true that a patient with h-IDS-D104V (z-IDS-D98V) has experienced the clinical manifestations of typical MPS II, including coarse facial features, skeletal deformities, hepatosplenomegaly, and valvular heart disease, with timely receipt of enzyme replacement therapy and hematopoietic stem cell transplantation. (2) Overexpression of z-IDS-R291H and z-IDS-T486I, which had partial enzyme activity, was not found to affect zebrafish embryonic development. These mutations were postulated to have a low correlation with MPS II development and may cause mild or no defects. In the current follow-up cases in our hospital, all h-IDS-R297H (z-IDS-R291H) and h-IDS-T500I (z-IDS-T486I) cases have no MPS II symptoms.

In this study, the enzymatic activity of IDS from proteins extracted from zebrafish embryos has been measured by using a fluorescent-tagged artificial 4-methylumbelliferone (4MU) substrate. The rate of fluorescence increase was directly proportional to enzyme activity. When comparing with the cost efficiency and the form of high-throughput screens, the fluorometric assay is not as good as the regular enzymatic assay. However, the benefit of its ability to predict developmental abnormalities is assured.

For the overexpression of three zebrafish mutated *ids* mRNAs corresponding to three known human mutated IDS effects on the embryonic development of zebrafish in this study, we observed three major defective phenotypes: (1) head defect, including smaller size and malformation of the craniofacial and pharyngeal arch; (2) eye defect, including smaller eyes and brain; and (3) body axis defect, including shortened somites and curved body. These phenotypes in zebrafish may correspond to the clinical manifestations in MPS patients, including skeletal deformities (dysostosis multiplex), eye defect, brain anomaly, and short stature.

In the current study, the abnormal embryonic development may be caused by the dominant negative effect of mutant IDSs on normal IDS activity. One possibility is to quantify the accumulated GAGs in the embryos to see whether there is any difference and whether this is the reason. However, there are technical limitations on GAGs detection and quantification in this situation. In the future, we will try to overcome the technique of detecting GAGs in zebrafish embryos and strengthen the proof of whether mutant forms of z-IDS in the embryo lead to GAG accumulation.

### 4.4. The Zebrafish Model Is a Fast and Simple Screening Platform for Evaluating the Pathogenicity of IDS Mutations Causing MPS II

In summary, this study provides a rapid and representative new method for studying zebrafish models in vivo, by overexpressing different mutant forms of z-IDS, to observe whether the zebrafish enzyme activity has an additive effect, and whether it leads to developmental defects. This can then help to understand the relationship between the mutant forms of z-IDS and the pathogenesis of MPS II in humans. In the present study, all nine of the IDS mutations were consistent with the clinical phenotypes of the human subjects from the MPS newborn screening programs in Taiwan, therefore, we believe this platform can be used as an important reference for medical professionals to assess the prognosis of MPS II subjects with different IDS mutations, and to provide them with the most timely and appropriate treatment at early disease stages.

## List of Abbreviations

MPS	mucopolysaccharidosis
IDS	iduronate 2-sulfatase
GAG	glycosaminoglycan

## References

[1] D’Avanzo F., Rigon L., Zanetti A., Tomanin R. (2020). Mucopolysaccharidosis Type II: One Hundred Years of Research, Diagnosis, and Treatment. Int. J. Mol. Sci..

[2] Lin H.-Y., Lin S.-P., Chuang C.-K., Niu D.-M., Chen M.-R., Tsai F.-J., Chao M.-C., Chiu P.-C., Lin S.-J., Tsai L.-P. (2009). Incidence of the mucopolysaccharidoses in Taiwan, 1984–2004. Am. J. Med. Genet. Part A.

[3] Khan S.A., Peracha H., Ballhausen D., Wiesbauer A., Rohrbach M., Gautschi M., Mason R.W., Giugliani R., Suzuki Y., Orii K.E. (2017). Epidemiology of mucopolysaccharidoses. Mol. Genet. Metab..

[4] Kim C.H., Hwang H.Z., Song S.M., Paik K.H., Kwon E.K., Bin Moon K., Yoon J.H., Han C.K., Jin D.-K. (2003). Mutational spectrum of the iduronate 2 sulfatase gene in 25 unrelated Korean Hunter syndrome patients: Identification of 13 novel mutations. Hum. Mutat..

[5] Kato T., Kato Z., Kuratsubo I., Tanaka N., Ishigami T., Kajihara J.-I., Sukegawa-Hayasaka K., Orii K., Isogai K., Fukao T. (2005). Mutational and structural analysis of Japanese patients with mucopolysaccharidosis type II. J. Hum. Genet..

[6] Neufield E.F., Muenzer J., Scriver C., Beaudet A.L., Valle D., Sly W.S. (2001). The mucopolysaccharidoses. The Metabolic and Molecular Bases of Inherited Disease.

[7] Chuang C.-K., Lin H.-Y., Wang T.-J., Huang Y.-H., Chan M.-J., Liao H.-C., Lo Y.-T., Wang L.-Y., Tu R.-Y., Fang Y.-Y. (2018). Status of newborn screening and follow up investigations for Mucopolysaccharidoses I and II in Taiwan. Orphanet J. Rare Dis..

[8] Chan M.-J., Liao H.-C., Gelb M.H., Chuang C.-K., Liu M.-Y., Chen H.-J., Kao S.-M., Lin H.-Y., Huang Y.-H., Kumar A.B. (2019). Taiwan National Newborn Screening Program by Tandem Mass Spectrometry for Mucopolysaccharidoses Types I, II, and VI. J. Pediatr..

[9] Lin H.-Y., Tu R.-Y., Chern S.-R., Lo Y.-T., Fran S., Wei F.-J., Huang S.-F., Tsai S.-Y., Chang C.-Y., Lee C.-L. (2020). Identification and Functional Characterization of IDS Gene Mutations Underlying Taiwanese Hunter Syndrome (Mucopolysaccharidosis Type II). Int. J. Mol. Sci..

[10] Moro E., Tomanin R., Friso A., Modena N., Tiso N., Scarpa M., Argenton F. (2010). A novel functional role of iduronate-2-sulfatase in zebrafish early development. Matrix Biol..

[11] Costa R., Urbani A., Salvalaio M., Bellesso S., Cieri D., Zancan I., Filocamo M., Bonaldo P., Szabò I., Tomanin R. (2017). Perturbations in cell signaling elicit early cardiac defects in mucopolysaccharidosis type II. Hum. Mol. Genet..

[12] Stainier D.Y.R., Raz E., Lawson N.D., Ekker S.C., Burdine R.D., Eisen J.S., Ingham P.W., Schulte-Merker S., Yelon D., Weinstein B.M. (2017). Guidelines for morpholino use in zebrafish. PLoS Genet..

[13] Bellesso S., Salvalaio M., Lualdi S., Tognon E., Costa R., Braghetta P., Giraudo C., Stramare R., Rigon L., Filocamo M. (2018). FGF signaling deregulation is associated with early developmental skeletal defects in animal models for mucopolysaccharidosis type II (MPS II). Hum. Mol. Genet..

[14] Westerfield M. (1995). The Zebrafish Book.

[15] Lin C.-Y., Lee H.-C., Chen H.-C., Hsieh C.-C., Tsai H.-J. (2013). Normal Function of Myf5 During Gastrulation Is Required for Pharyngeal Arch Cartilage Development in Zebrafish Embryos. Zebrafish.

[16] Lin C.Y., Wu C.L., Lee K.Z., Chen Y.J., Zhang P.H., Chang C.Y., Harn H.J., Lin S.Z., Tsai H.J. (2019). Extracellular Pgk1 enhances neurite outgrowth of motoneurons through Nogo66/NgR-independent targeting of NogoA. eLife.

[17] Vafiadaki E., Cooper A., Heptinstall L.E., Hatton C.E., Thornley M., Wraith J.E. (1998). Mutation analysis in 57 unrelated patients with MPS II (Hunter’s disease). Arch. Dis. Child..

[18] Lin S.-P., Chang J.-H., Lee-Chen G.-J., Lin D.-S., Lin H.-Y., Chuang C.-K. (2006). Detection of hunter syndrome (mucopolysaccharidosis type II) in Taiwanese: Biochemical and linkage studies of the iduronate-2-sulfatase gene defects in MPS II patients and carriers. Clin. Chim. Acta.

